# Considerations surrounding remote medicolegal assessments: a systematic search and narrative synthesis of the range of motion literature

**DOI:** 10.1111/ans.16841

**Published:** 2021-04-23

**Authors:** Peter Steadman, Dianne Sheppard, Janette Henderson, Brett Halliday, Ian Freckelton

**Affiliations:** ^1^ Office of the Chief Medical Office MedHealth Melbourne Victoria Australia; ^2^ Research & Innovation MedHealth Melbourne Victoria Australia; ^3^ Clinical Business Services MedHealth Melbourne Victoria Australia; ^4^ Barrister Castan Chambers Melbourne Victoria Australia; ^5^ Department of Law and Psychiatry University of Melbourne Melbourne Victoria Australia

**Keywords:** arthrometry, articular, assessment, evidence, expert, legal, medicine, orthopaedic surgery, range of motion, telemedicine

## Abstract

Remote telehealth practices were forced to advance 10 years in a few short weeks in March 2020 due to the onset of a global pandemic. In the sphere of non‐clinical medicine, a dramatic element of uncertainty entered the psyche of doctors and lawyers in relation to the validity of remote or *virtual* independent medical examination (vIME). This paper considers the key issues surrounding the virtual assessment of clients for medicolegal purposes. Our main hypothesis was that, within certain defined parameters, the vIME technique can deliver reliable and accurate assessments. To explore this, a systematic literature search focusing on advanced device‐based range of motion measurement was conducted, along with an historical snapshot of observation‐based range of motion measurement considering application to remotely performed IME. While some specialists are of the view that observational measurement may be applied reliably to some joints when conducted by experienced orthopaedic surgeons, evidence for this is scant. The results, instead, support the notion of using *task substitution*, that is specialists appropriately assisted in conducting vIMEs by musculoskeletal trained allied health practitioners, regardless of the measurement tool, for permanent impairment assessments. Moreover, self‐performed examinations by injured individuals using advanced technology are not reliable in this setting. Our final contention is that remote examinations with limited clinical assessment have utility for legal matters, such as the assessment of causation of injury, treatment advice or approvals and fitness for pre‐employment tasks or safe variations, with objective clinical adjunct support such as Picture Archiving and Communication System‐based modern radiology systems.

## Graphical abstract

This paper addresses a previously unexplored gap in considering the potential for remote or virtual independent medical examinations (vIME), and is a step towards demonstrating that best practice vIME techniques, within certain defined parameters, can deliver reliable and accurate assessments. The key elements of this manuscript include: a systematic literature search focusing on advanced device‐based range of motion measurement, and an historical snapshot of observation‐based measurement considering application to remotely performed IME.

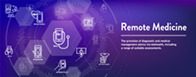

In clinical musculoskeletal medicine, range of motion (ROM) tells us many important pieces of information about a patient's condition. By observing the movements of different body parts, we are able to determine the primary physical challenges following an injury and measure change objectively over time.

There is a barrier of distance affecting regional Australia when it comes to medical care. Telehealth represents a contemporary option that complies with travel restrictions and need for safe social distancing stemming from the COVID‐19 pandemic. Telehealth practices were forced to advance 10 years in a few short weeks in March 2020,^1^, ^2^ suddenly expanding into mainstream use in clinical practice including the release of a Medicare item number. As a result, an element of uncertainty surrounded the validity of new, remote forms of independent medical examinations (IME). While already utilized extensively in psychiatry examinations, musculoskeletal clinicians who routinely use physical findings to assess permanent impairment were caught underprepared for the forced change. This created a need to determine whether telehealth presents a reliable modality for assessing physical loss and reviewing patient outcomes. This paper looks at the issues surrounding the formal assessment of injured individuals in the medicolegal setting. In this sector, clinicians complete ‘independent medical examinations’ to examine injured individuals and, amongst other things, convert physical signs into a percentage to determine loss. This figure allows the insurers, the legal profession, courts and tribunals to determine the level of financial compensation related to the physical injury.

The primary objective of this paper is to examine this topical, contentious issue and come to some conclusions to assist clinicians using telehealth for medicolegal purposes and determine the most reliable method(s) for performing clinical examinations that will also satisfy legal requirements. We use terminology recently adopted to reflect this change in examination technique, i.e. virtual IME (vIME).

Measurement of ROM has been in ongoing development for many decades (Fig. S1); its validity using visual estimation (VE) alone or goniometric methods (particularly the universal goniometer, UG) has been well established.^3^ We also know that the reliability of ROM measurement using goniometry is influenced by many factors, including instrumentation, procedures, joint actions and body regions.^3^, ^4^ More recently, evidence is accumulating that supports using smartphone applications to measure ROM in place of traditional goniometry techniques.^5^ While the timeline (Fig. S1) provides a useful historical snapshot of ROM assessment over the last 120 years, the analysis of the most recent technological advances *over the past 15 years*, presented in the body of this paper, allows an evaluation of whether new technologies are able to advance the reliability of vIME assessments.

Due to the lack of evidence relevant to the vIME setting, hypotheses were formed by integrating the evidence from the general orthopaedic setting with the clinical experience of two orthopaedic surgeons (PS and BH). To assist clinicians using telehealth for medicolegal purposes, we extrapolated the findings to the vIME setting, formulating scenarios upon which the legal system can rely.

Our key hypothesis is that the current evidence is unlikely to support performing *unassisted* examinations within the vIME setting for *permanent impairment assessment*, where rigorous physical examination and precise measurements are required (with reference to the American Medical Association (AMA) Guides' permanent impairment textbook tables and algorithms). We predict that the evidence will suggest that *unassisted* vIME, where the finer aspects of the examination are less critical, should be limited to certain types of assessment, such as causation of injury, treatment advice and return to work assessment.

We examined the conditions under which observational goniometry (i.e. VE), traditional goniometry and modern manual or electronic goniometrical devices and smartphone Apps are consistently accurate (valid and reliable). A key consideration is whether there is adequate evidence that the method of measurement can be performed reliably in the vIME setting with clinician guidance, *with or without* the assistance of a musculoskeletal‐trained allied health practitioner (AHP) on site with the injured individual. We examined whether certain modern technologies are suitable to be used during assessments of the injured individual alone in a remote location (i.e. home‐based vIME assessment), or whether such tools and technologies require the physical presence of an AHP with musculoskeletal knowledge and positional training to allow the IME practitioner to achieve the required measurement standards of the permanent impairment guides.

## Methods

### Context and setting of the study

Standard medical examination for orthopaedic conditions requires musculoskeletal measurement to determine patient recovery. Similarly, the extent of permanent impairment is determined by converting a patient's musculoskeletal injury into a ‘percentage of bodily function loss’ to help lawyers, courts and tribunals determine financial compensation. It is the role of doctors to determine the ROMs and plot these into the predetermined ROM algorithms using the AMA guides.^6^ The AMA assessment guides form the essential text for permanent impairment assessment in Australia. Initially, a consensus document developed in the USA, the AMA Guides are now widely adopted by almost every jurisdiction in Australia to assess permanent impairment either directly or in some modified form.^6^

### Search strategy and selection of studies

A systematic search of papers published from January 2005 to end of May 2020 and limited to English was undertaken within the MEDLINE‐Ovid database.

The search terms and their combination were:

- range of motion/ or range of movement/ or goniomet*/ or visual estimation/ or visual inspection/ or visual assessment/.mp

AND

- remote/ or virtual/ or video/ or mobile application/ or smartphone/ or telehealth/ or telemedicine/ or teleorthopedic/ or telerehabilitation/.mp

AND

- reliability/ or valid*/ or accuracy/ or quality/.titl

All titles were then scanned for relevance and abstracts read where eligibility criteria (Data S1) were met. Papers were excluded at abstract stage due to a primary focus on *other types* of quality or functional movement‐based assessments (i.e. a lack of focus on joint ROM assessment). Commonly excluded were papers examining the reliability, validity or accuracy of other types of technologies, including 3D motion analysis systems, and any commentary or opinion‐pieces. Full text was sought for those articles still remaining (see Fig. S2).

A secondary search strategy captured the historical perspective of ROM assessment methods reporting on the accuracy and reliability of VE to assess ROM. Papers discussing the emergence of the ‘telehealth’‐specific methodologies were reviewed. The secondary search involved a reference list search of all primary search‐identified full‐text papers (keywords: surgeon, orthopaedic, visual + inspection, estimation, observ*, goniomet*, joint angle measurement, clinomet*, telemedicine/teleassessment/telerehabilitation/telehealth, mobile app*, smartphone).

## Results

### Search results

The primary MEDLINE database search identified a total of 142 articles. After excluding those not meeting inclusion criteria (*n* = 72), the abstract review of 70 studies resulted in 67 full‐text articles being sourced.

A further 82 studies were identified during secondary reference list scanning, including 25 relevant to addressing the hypotheses, and others of potential historical relevance (Fig. S1). The final full‐text eligibility screen excluded those not of central relevance to the vIME assisted or unassisted context, leaving a total of 55 articles for inclusion.

### Data extraction results

The data tables (Tables [Link], [Link]) present key information from the final set of papers that were deemed relevant for two specific scenarios:

1. Observational only or other ROM measurement (e.g. virtual goniometry) that could be conducted *without* a musculoskeletal‐trained AHP on site (untrained, e.g. family member, assistance permitted)
2. Assisted measurement with a musculoskeletal‐trained AHP (not the examining specialist) on site with the patient, using a measurement tool (goniometer or App).

Information extracted and presented includes conditions or moderating factors shown by the results to influence the accuracy/reliability as well as limitations and information pertaining to the suitability of using the tool(s) in the vIME / telehealth setting.

**Scenario 1:** *Observational only* methods of ROM assessment relevant to the vIME setting performed *without* the assistance of a musculoskeletal (MSK)‐trained AHP.

The data presented in Table S1 demonstrate that observational goniometry or ROM measured by VE is not consistently reliable (references 1–7 from Table S1). This did not differ across the type of joint. Therefore, although logistically suitable for the vIME environment, there is a lack of evidence for the use of observational goniometry or VE alone when precise measurements are required.

Six studies were identified that presented preliminary evidence of validity for tools (either a photographic‐based ROM App, or a goniometer used in a telehealth setting at the clinician end) suitable for measuring ROM in the vIME (telehealth) setting. Reliability was not consistently reported to be high. Although suitable for the telehealth environment, it should be noted that all but one of the techniques (reference 8 from Table S1) required the use of photographs (or still images of video footage) and the identification of landmarks (post‐examination) to maximize accuracy. All required correct positioning and aligning of the patient with the camera. Preliminary evidence was also found for the reliable use of internet‐based goniometry when used with standardized instructions and positioning. However, more rigorous research is required to confirm the reliability and accuracy of measurements using the internet‐based goniometer for permanent impairment assessments.

There is preliminary evidence that sophisticated App technologies that use in‐built sensors may facilitate measurement of ROM, at least for certain joints, for patients in the vIME setting *without* the assistance of an onsite MSK‐trained AHP (Table S1). The most promising is perhaps the ‘Dorsiflex’ App used to measure ankle ROM using an iPhone 8,^9^ reported to be easy to use, and not requiring landmark identification or sophisticated set‐up.

**Scenario 2:** *Assisted measurement* using modern methods of ROM assessment with presence of a MSK‐trained AHP on site with the patient.

Table S2 shows a selection of the most relevant studies from the past 15 years examining the reliability and validity of modern tools and instruments measuring ROM,^5^, including, for example the digital goniometer, specialized / adapted goniometers, computerized goniometers, as well as a wide variety of smartphone Apps (These studies (Table S2) largely examined the validity and/or reliability of ROM measurement *for a particular joint*. As some specific joints, positions or movements are more complex (reference 15 from Table S2) or generally result in higher absolute error, it is important not to generalize findings to other joints). These tools and devices may be appropriate for the vIME environment, but would require onsite MSK‐trained AHP assistance. We separated the tilt‐ and motion‐based (i.e. *non‐photographic* inclinometer and/or accelerometer) smartphone Apps (references 6, 9 from Table S1; references 1–25 from Table S2), from those papers reporting reliability and validity of photographic‐based Apps (Our focus was on Apps with in‐built tilt (inclinometer) and motion (accelerometer) sensors; we purposefully only reported a few, randomly selected, studies of photographic‐based App technologies) (references 20, 21, 26–30 from Table S2). A selection of other types of novel tools or instruments was also included where deemed potentially relevant for the vIME context (references 31–37 from Table S2).

Table S2 presents the substantial body of literature demonstrating the validity, and often superior reliability, of the more modern ROM instruments when performed by an examiner with musculoskeletal knowledge and training. Few studies were identified that *directly* contrasted the reliability and validity of these new instruments with those of observational goniometry (Table S2). Two studies are worthy of further mention. Werner *et al*. (reference 30 from Table S2) showed superior inter‐rater reliability for smartphone App shoulder ROM measurements compared to both standard goniometer and VE measurements (across a group of examiners with variable skill levels). Unfortunately, intra‐observer reliability was not measured or compared across methods. ‘Surgeon visual estimation’ was also recently found only to be accurate for the *forearm rotation* movement when measuring elbow ROM; however, the two other techniques of elbow ROM measurement examined by this study were also found to have inadequacies relative to the goniometer gold standard (reference 6 from Table S1).

These results (Table S2) illustrate that clinicians are now able to use modern smartphone applications reliably to measure ROM in the face‐to‐face clinical^5^), or in the VIME setting *when aided by an allied health practitioner with musculoskeletal training on site with the patient* (Of note, older smartphone models (i.e. iPhone 4 or prior) may not have in‐built technology (i.e. inclination sensors or accelerometer technology) required by the non‐photographic ROM Apps). The majority of studies reported also recommend certain conditions required to maximize accuracy, reliability and/or validity of ROM measurement when using these instruments. Anatomical expertise or experience to facilitate standardized instrument placement (position, distance, standardization *etc*.), and/or knowledge of anatomy (reference 6 from Table S1; 1–4, 8–14, 17–21, 23, 25, 26, 30, 33, 34, 37, 38 Table S2) were most commonly required. For some instruments there were indications that rigorous standardized procedures requiring assessor training were required to maximize reliability (references 15, 27, 29, 31, 36 from Table S2).

One final limitation presenting an insurmountable challenge in the vIME setting is the requirement of (sometimes) specialized equipment to aid placement of the device or to hold part of a body in the correct position during the measurement. This often requires substantial initial set‐up time and effort (references 2, 5, 7, 9, 12, 14, 16, 20, 22, 28, 32, 35 from Table S2).

## Discussion

Face‐to‐face clinical examination using goniometry represents the ‘gold standard’ in ROM assessment. Evidence reported here challenges the uniform necessity for the in person element, echoing a recent systematic review.^5^ Evidence supports the use of modern smartphones with ROM Apps instead of more traditional goniometers to measure joint ROM in the broader orthopaedic setting.^5^ The wide diversity in the Apps utilized in the studies reported by this review and that of Keogh *et al*.,^5^ suggest that the orthopaedic or MSK clinician has multiple options when selecting an App for measuring a particular joint ROM. Moreover, the most recently available non‐photographic smartphone ROM App technology also seems to have potential for use in the vIME context (references 6, 9 from Table S1; references 1, 8, 11, 12, 14, 17 from Table S2).

When considering the application of these ROM assessment methods within the vIME environment, our key hypothesis was supported, that is there was a lack of evidence to support performing *unassisted* examinations for *permanent impairment assessment* where rigorous physical examination and precise measurement are required. Observational goniometry or VE demonstrated inadequate reliability, and the evidence for emerging technologies was not yet convincing enough for these types of assessments. vIME would, however, be adequate for performing clinical examinations requiring a less thorough physical examination, that is when limited to certain types of assessment. For cases such as causation of injury, treatment advice and return to work assessment, *unassisted* clinical examination is valid in the vIME environment provided that the examination is performed remotely by an experienced musculoskeletal practitioner such as an orthopaedic surgeon. ROM measurement via observational goniometry may be satisfactory if limited to certain joints. The knee, ankle, wrist, shoulder and elbow joints should be able to be assessed within the vIME setting in readily observable planes of movement. This would, however, require further specific research to verify the validity and reliability.

We have identified the specific circumstances that are likely to maximize the accuracy and reliability of ROM measurement in the vIME setting. There is convincing evidence *against* the clinician using observational ROM measurements *in isolation*. There is also a lack of evidence to support the injured individual being able to use an App to measure ROM reliably in the absence of MSK training or the presence of an assistant (trained or untrained), despite recent technological advances. There is no doubt, however, that such Apps will continue to evolve with the development of further smart phone technology. Moreover, in the instance of permanent impairment assessments, there is the additional risk of unreliability if the claimant is required to facilitate their own assessment in which they have a potential conflict of interest.

Our results have provided support for using modern manual or electronic devices, or ROM Apps in the vIME setting *when assisted by a musculoskeletal‐trained AHP*, even for permanent impairment assessment where rigorous physical examination and precise measurement are required. The accumulated evidence suggests that a MSK‐trained AHP (e.g. physiotherapist or accredited exercise physiologist) using any valid goniometric instrument or App would be likely to reach the same conclusions as a highly skilled surgeon, assuming familiarity with the device underpinning the process of successful measurement. In this circumstance, the MSK‐trained AHP would conduct the ROM assessment in the presence of the claimant under direct instruction of the clinician observing the assessment remotely.

The vIME environment does have some limitations that are impossible to overcome. One critical aspect unique to the IME setting is the somewhat subjective assessment of ‘participation’ by the injured individual, which is challenging to observe even in the gold standard face‐to‐face circumstances. Although a MSK‐trained AHP can assist with the measurement aspect, they may not have the training to appreciate these subjective nuances that experienced medicolegal specialists can observe and evaluate. For this reason, MSK‐trained AHP assisted vIME may not be appropriate for permanent impairment judgements where, for example, a neurological examination is required that could lead to an injury category change by some percentage points. Moreover, spine conditions with radiculopathy present a limitation to virtual examination that either requires the examiner to use a different diagnostic technique or a different model for permanent impairment assessment.

Our results have provided sufficient evidence that assisted examination represents the best available option when telehealth is required. In this case performed by a suitably trained (allied health) MSK practitioner supervised remotely by the IME examiner in a COVID‐19‐safe protocol setting. The importance of such assistance is emphasized in most of the examined literature demonstrating that the patient's position during the examination is critical to maximize the accuracy of angular measurements.

### Clinical implications and recommendations

The accumulation of evidence supports that *assisted* remote examination *will* represent the ‘gold standard’ for Virtual IMEs. When directed assistance is provided by a well‐trained MSK health practitioner using a valid measurement tool, this review demonstrates that those measurements have satisfactory reliability for forensic purposes.

‘Normal’ clinical practices seem a long way off in the midst of this COVID‐19 global pandemic. Potentially placing a qualified assistant at risk of exposure at the patient‐end raises professional ethical considerations surrounding ‘task substitution’; however, we are fast becoming accustomed to employing a COVID‐19 safe environment using detailed safety protocols to avoid exposing health practitioners to unnecessary risk.

It is inappropriate to allow injured individuals to participate in their own assessment of loss of physical function using a smartphone measurement device in a system within which there are positive compensation benefits associated with poor participation. With this in mind, the evidence presented suggests that the vIME is likely to be a valid and viable setting for most non‐impairment categories to achieve reliable, accurate and timely assessments of legal importance such as causation, ongoing treatment, or fitness to return to pre‐injury duties. Investigative diagnostic adjuvants such as PACS radiology (i.e. radiological tests performed locally, viewed remotely by the practitioners with consent) also reduce the reliance on face‐to‐face physical examination.

Inevitably, the reliability of the vIME will be tested by cross‐examination. Adherence to robust protocols and retention of carefully documented assessment records will be vital for confidence in the reliability of results that are presented.^7^ The fact that a vIME will be conducted only in limited circumstances will be important for how results are received. There will be occasions when both the orthopaedic assessor and the AHP will be required for cross‐examination. Provided that there is clear allocation of assessment responsibilities, formulation of and compliance to vIME protocols, and retention of full assessment documentation, this should not result in adverse evidentiary determinations as to admissibility or probative value.

In conclusion, there is sufficient evidence to suggest that assisted medical examination in a vIME setting represents a satisfactory modality for permanent impairment assessments with certain limitations as discussed. The next step is the development of detailed guidelines and protocols to maximize both efficiency and consistency of methodology.

## Author Contributions

**Peter Steadman:** Conceptualization; supervision; writing‐original draft; writing‐review & editing. **Dianne Sheppard:** Data curation; formal analysis; investigation; methodology; project administration; writing‐original draft; writing‐review & editing. **Janette Henderson:** Data curation; formal analysis; project administration; resources; validation; visualization. **Brett Halliday:** Writing‐review & editing. **Ian Freckelton:** Writing‐review & editing.

## Conflicts of interest

PS, DS, JH and BH are all employees of MedHealth Pty Ltd. in Australia. MedHealth is a national provider of IME.

## Supporting information


**Data S1.** Eligibility criteria.

**Figure S1.** A detailed timeline that provides an historical ‘snapshot’ of the variety of methods used to assess ROM over the last 120 years.

**Figure S2.** PRISMA flow chart of primary and secondary literature search, screening, selection and inclusion.

**Table S1.** Studies examining the reliability and validity of ROM^1^ assessments conducted with *measurement tools or App that do NOT require* musculoskeletal‐trained allied health practitioner (AHP) assistance, including visual estimation.

**Table S2.** ROM techniques and technologies (goniometers or Apps)^1^ with potential to be administered in the vIME setting with assistance of a musculoskeletal‐trained allied health practitioner (AHP) on site.

## References

[1] ABC News . *Move to Telehealth Is Here to Stay After Coronavirus*. 2020. [Cited 5 May 2020.] Available from URL: https://www.abc.net.au/news/2020-05-05/move-to-telehealth-is-here-to-stay-after-coronavirus/12212680

[2] Verduzco‐Gutierrez M , Bean AC , Tenforde AS , Tapia RN , Silver JK . How to conduct an outpatient telemedicine rehabilitation or prehabilitation visit. PM R 2020; 12: 714–20. 32297458 10.1002/pmrj.12380

[3] van Rijn SF , Zwerus EL , Koenraadt KL , Jacobs WC , van den Bekerom MP , Eygendaal D . The reliability and validity of goniometric elbow measurements in adults: a systematic review of the literature. Shoulder Elbow 2018; 10: 274–84. 30214494 10.1177/1758573218774326 PMC6134535

[4] Gajdosik RL , Bohannon RW . Clinical measurement of range of motion. Review of goniometry emphasizing reliability and validity. Phys. Ther. 1987; 67: 1867–72. 3685114 10.1093/ptj/67.12.1867

[5] Keogh JWL , Cox A , Anderson S *et al*. Reliability and validity of clinically accessible smartphone applications to measure joint range of motion: a systematic review. PloS One 2019; 14: e0215806. 31067247 10.1371/journal.pone.0215806 PMC6505893

[6] Cocchiarella L , Andersson GBJ . Guides to the Evaluation of Permanent Impairment, 5th edn. Chicago, IL: American Medical Association, 2001.

[7] Freckelton I . Expert Evidence: Law, Practice, Procedure and Advocacy, 6th edn. Sydney: Thomson Reuters, 2019.

