# Supplementary material for: Considerations surrounding remote medicolegal assessments: a systematic search and narrative synthesis of the range of motion literature
Source: ANZ J Surg. 2021 Apr 23;92(1-2):46–50. doi: 10.1111/ans.16841 (PMC9291801; doi:10.1111/ans.16841)
Supplement: Supplementary file 1 — Data S1. Eligibility criteria. [file ANS-92-46-s003.docx]

# Doc S1: Eligibility criteria

i) Study design

i.e. Controlled trials contrasting ROM assessment tools / techniques; validity, reliability of ROM assessments; particularly those conducted in telehealth setting where available. Both healthy and clinical populations were deemed relevant and so both included.

ii) Peer-reviewed

iii) Specific type(s) of ROM measurement and tool(s) i.e. exclude papers where only assess quality of movement or functional movement patterns. Include use of goniometer, inclinometer etc and smartphone App imitating the goniometer
