# Supplementary material for: Considerations surrounding remote medicolegal assessments: a systematic search and narrative synthesis of the range of motion literature
Source: ANZ J Surg. 2021 Apr 23;92(1-2):46–50. doi: 10.1111/ans.16841 (PMC9291801; doi:10.1111/ans.16841)
Supplement: Supplementary file 2 — Figure S1. A detailed timeline that provides an historical ‘snapshot’ of the variety of methods used to assess ROM over the last 120 years. [file ANS-92-46-s001.docx]

**Timeline A: 1900 – 1987**
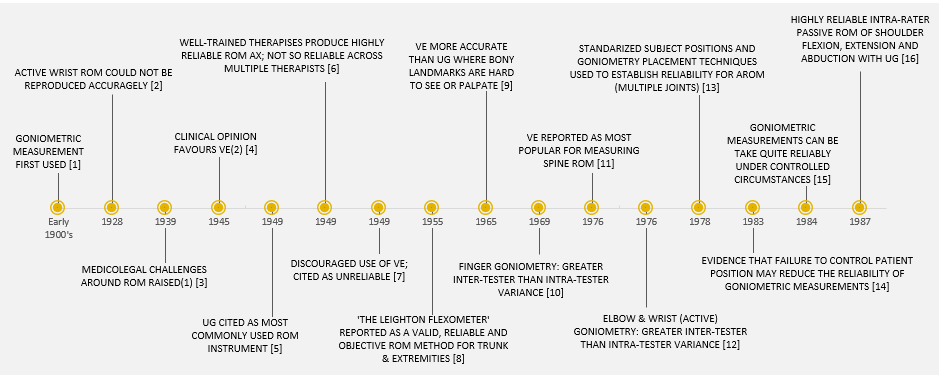


**Timeline B: 1991 – 2019**
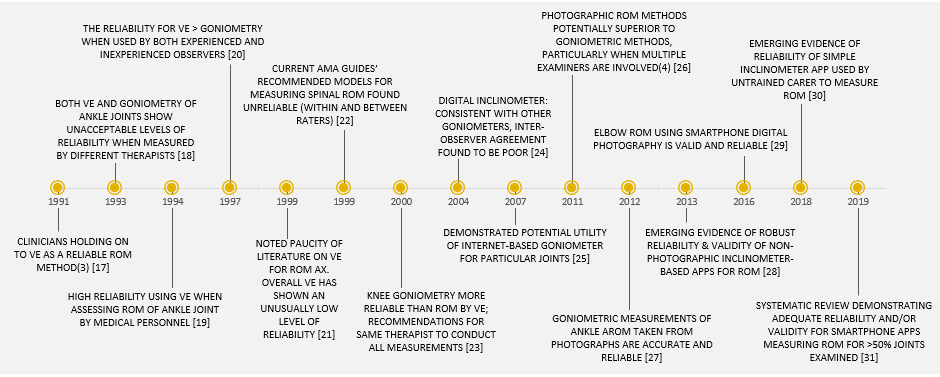


**Figure S1:** A detailed timeline that provides an historical ‘snapshot’ of the variety of methods used to assess ROM over the last 120 years

| Footnotes | | |
| --- | --- | --- |
| YEAR | EVENT | FOOTNOTE |
| **1939** (1) | Medicolegal challenges around ROM raised^^[[1]](#footnote-1)^^ [3] | i.e. specify methods of ROM measurement and movements, common nomenclature and definitions, establishment of norms. |
| **1945** (2) | Clinical opinion favours VE^^[[2]](#footnote-2)^^ [4] | ^2^ Quote: […no amount of] “objective measurement could be substituted for the subjective estimation of an experienced surgeon”. |
| **1991** (3) | Clinicians holding on to VE as a reliable ROM method^^[[3]](#footnote-3)^^ [17] | ^3^ Personal commentary suggested ‘that there was enough evidence that visual estimation was not reliable and that therapists should no longer be using it as part of their assessment of ROM’. |
| **2011** (4) | Photographic ROM methods potentially superior to goniometric methods, particularly when multiple examiners are involved^^[[4]](#footnote-4)^^ [26] | ^4^ Patient positioning, photographic technique and care in obtaining the limits of extension and flexion remain important. |
|  |  |  |
| Legend | | |
| ACRONYM | MEANING | |
| VE | Visual Estimation |  |
| UG | Universal Goniometry |  |
| ROM | Range of Motion |  |
| AROM | Active Range of Motion |  |

1. [↑](#footnote-ref-1)
2. [↑](#footnote-ref-2)
3. [↑](#footnote-ref-3)
4. [↑](#footnote-ref-4)
