# Supplementary material for: Considerations surrounding remote medicolegal assessments: a systematic search and narrative synthesis of the range of motion literature
Source: ANZ J Surg. 2021 Apr 23;92(1-2):46–50. doi: 10.1111/ans.16841 (PMC9291801; doi:10.1111/ans.16841)
Supplement: Supplementary file 3 — Figure S2. PRISMA flow chart of primary and secondary literature search, screening, selection and inclusion. [file ANS-92-46-s002.docx]

Identification

Included

Screening

Eligibility #1

Records identified through database searching
n=1404

Additional records identified through citation tracking
n=4

Records screened after duplicates removed and abstract screening

n=142

Records excluded
n=75

Full text assessed for eligibility
n=67

Secondary search
inclusions n=25

Studies included from both searches
n=92

Studies included in data analysis

n=55

Eligibility #2

Full text articles excluded
n=37

**Figure S2:** PRISMA flow chart of primary and secondary literature search, screening, selection and inclusion
