# Supplementary material for: Considerations surrounding remote medicolegal assessments: a systematic search and narrative synthesis of the range of motion literature
Source: ANZ J Surg. 2021 Apr 23;92(1-2):46–50. doi: 10.1111/ans.16841 (PMC9291801; doi:10.1111/ans.16841)
Supplement: Supplementary file 4 — Table S1. Studies examining the reliability and validity of ROM1 assessments conducted with measurement tools or App that do NOT require musculoskeletal‐trained allied health practitioner (AHP) assistance, including visual estimation. [file ANS-92-46-s004.docx]

**Table S1**: Studies examining the reliability and validity of ROM assessments^[[1]](#footnote-1)^ conducted with *measurement tools or App that do NOT require* musculoskeletal-trained allied health practitioner (AHP) assistance, including visual estimation

| *Author(s), year* | Joint(s) | Movement & Position | ROM Instrument(s) | Reliability & validity | Moderating Factors | Limitations, observations, information RE usability in VIME setting |
| --- | --- | --- | --- | --- | --- | --- |
| *3. Hayes K, Walton JR, Szomor ZR et al.(2001)* | shoulder | Seated upright, feet supported on a foot stool; flexion, abduction and external rotation | VE; visual estimation, universal goniometry, still photography, stand and reach test, tape measured distance | Fair to good intra-rater reliability was reported for three shoulder movements assessed by VE & goniometry. Absolute intra-rater reliability (SEMs) did not reach the 'good threshold standard' (Keogh) for UG or VE. | N/A | Additional evidence that VE alone is not sufficiently reliable for use within the clinical setting. |
| *7. Youdas JW, Bogard CL, Suman VJ. (1993)* | ankle | Ankle dorsiflexion; ankle plantar flexion; prone on table or mat; active | VE; visual estimation vs universal goniometer | 'Good degree of reliability' reported when using UG; inadequate reliability of VE | N/A | VE showed inadequate reliability; UG would require AHP assistance. |
| *1. Colaris J, van der Linden M, Selles R et al.(2010)* | forearm | Standing; pronation and supination (elbow 90 ^o^, forearm mid-position, wrist neutral) | VE; visual estimation vs universal goniometer | Better absolute reliability (lower measurement error) for goniometer compared to VE. Reliability of goniometer (inter- and intra-rater) was excellent (Keogh criteria). | Reliability higher when same rater was used (inter-rater reliability lower) for both methods. | Note - paediatric cohort with previously sustained forearm fractures. Using the goniometer would require AHP assistance in the VIME setting. |
| *5. Hirsch, B.P., Webb, M.L., Bohl, D.D. et al.(2014)* | spine (cervical) | Seated, head in neutral position. flexion-extension, lateral bending, and rotation | VE; Visual estimation vs electrogoniometer before and after 'ROM training' | Although reasonable inter-rater reliability was demonstrated for VE, significant errors (relative to electrogoniometer as criterion) in CROM were found in all planes (Keogh criteria not relevant) | ROM training' for clinicians improved accuracy of visual estimation in 3 planes, but this improvement was not sustained at 1 month for all planes | Findings suggest that visual estimation alone is *not reliable enough for the determination of impairment in the medicolegal setting*. |
| *2. Croxford P, Jones K, Barker K. (1998)* | ankle | Full active range of ankle dorsiflexion was measured. Testers (12 experienced orthopaedic physiotherapists) used their own standard positioning and examination techniques. | VE; Universal goniometer (UG) vs visual estimation | Excluding outliers, maximum measurement error was 6 ^o^ using a UG, and 11 ^o^ using VE. The extent of error for both techniques was clinically unacceptable. Results in favour of using a goniometer. | Evidence that NOT requiring raters to adhere to established, standardised protocols contributed to measurement error; i.e. lack of standardisation of patient positioning, alignment of instrument (UG) and movement start position. | Use of goniometry requires the presence of an AHP in the telehealth setting. |
| *4. Higashi M; Shofler D; Manji K et al. (2017)* | Foot - hallux abducto valgus (Intermetatarsal) | 1st inter-metatarsal joint (no information on movement / position) | VE; (examining effect of angle & experience level) | Reliability of visual estimation not established. VE differed from manual measurements by 3.28^o^ (SE 1.56 ^o^). | Accuracy and inter-rater reliability of VE measurements improved with experience, though still not to acceptable levels; accuracy was worse for larger angles | IM angle visually estimated from radiographs |
| *10. Werner BC; Holzgrefe RE; Griffin JW et al. (2014)* | shoulder | Abduction and forward flexion measured standing position; external and internal rotation measured supine | App Type 1; Smartphone clinometer application vs visual estimation and goniometer | Excellent inter-rater reliability and average validity reported for the smartphone clinometer (substantially higher than VE); Intra-rater reliability not examined (Keogh) | No evidence for 'experience' (skill level) influencing reliability of ROM assessments made with the smartphone clinometer | Smartphone held against the patients' arm (varying positions) and measurements read from the screen. Shoulder ROM likely to be more challenging via telehealth with assistance of unskilled carer. Likely to require AHP presence for reliable ROM measurements. |
| *6. Vauclair F, Aljurayyan A, Abduljabbar FH et al. (2018)* | elbow | Flexion, extension, pronation, supination (active end ROM), in sitting position for all devices. | App Type 1; Smartphone App (Clinometer) vs UG, X-Ray & (Surgeon) visual estimation. | Excellent absolute validity (Keogh); Relative validity reported to be 'not as good as expected' (vs UG); reliability not measured | N/A | App was found to be most highly associated with criterion when measuring the complete arc of motion. *Further research required to confirm reliability. Presence of an untrained carer likely adequate to allow the use of the App in telehealth, i.e. clinician to instruct how to assume the correct position, and a carer to position the smartphone and read the screen. |
| *11. Pourahmadi MR; Ebrahimi Takamjani I et al. (2017)* | wrist (active ROM) | Seated standard position active wrist ROM, volar/dorsal alignment technique for: flexion, extension, radial deviation & ulnar deviation. | App Type 1; iPhone App (G-pro) comparison for concurrent validity with universal goniometer | Good to excellent intra- and inter-rater reliability (Keogh); although absolute validity was high, relative validity did not quite meet the 'substantial' Keogh threshold. | N/A | Measurement of wrist ROM obtained after strapping the iPhone to the patients' hand (controlling placement and preventing slippage). AHP assistance required in telehealth setting for both devices. |
| *12. Mejia-Hernandez K, Chang A, Eardley-Harris N et al.(2018)* | shoulder | Seated, active forward flexion: total abduction, active abduction, and passive abduction. Rotation (internal, external, active, passive) of shoulder in supine. | App Type 1; Inclinometer based App [GetMyROM], photograph-based [DrGoniometer] App vs UG and Clinician visual estimation. | Adequate validity & excellent inter-rater reliability demonstrated for both smartphone Apps (intra-rater reliability not measured) (Keogh); high reliability also shown for visual estimation for the 2 highly trained raters with several years clinical experience | N/A | iPhone (5S model) attached to patients' arm at various positions using an arm-band when using the inclinomer-based App (could be administered by untrained carer); markers on landmarks were required for the photographic-based App; AHP presence for telehealth setting required for complexity of shoulder ROM positioning and landmark identification for photo-based App. |
| *9. Balsalobre-Fernandez C; Romero-Franco N et al. (2019)* | ankle | Weight-bearing lunge; ankle dorsiflexion | App Type 1; Dorsiflex' iPhone app (iPhone 8 with in-built sensors) vs Professional digital inclinometer | App reported to be highly valid, reliable and accurate (reliability at same level as digital inclinometer). | Need to consider type of smartphone used | Larger screen-devices not tested; Need a modern smartphone device with (inbuilt) inclination sensors (iPhone 8 used); Ease of use similar to that of inclinometer, with no additional professional equipment required; app demonstrates correct placement (can be self-administered). |
| *13. Cuesta-Vargas AI & Roldan-Jimenez C (2016)* | shoulder/ arm abduction angle | Abduction measured standing. Shoulder height measured, so Smartphone could be positioned at the same height | App Type 2; Smartphone **image-based** goniometer app (mROM) | Good intra-rater and inter-rater reliability was found for the App, but NOT for the healthy group alone. No evidence was found for criterion validity (using unusual image-based criterion). Keogh criteria applied. | To minimise error instructions are required to specify smartphone placement, patient positioning, and locating landmarks | Not generalisable yet to other joints or other shoulder movements (specific to arm abduction angle); photograph or image-based apps required standardised instructions for positioning to minimise error. Photographs in position(s) are saved to device. |
| *8. Cabana, F. et al. 2010* | knee | flexion and extension; subject was perpendicular to the camera and UG | Other; Telerehabilitation vs face-to-face modes of assessment (including ROM); UG used at clinician end | Only inter-rater reliability was measured, with agreement between face-to-face and telerehabitation rated as 'good', with highest mean difference of 6.3^o^ for knee flexion. | Both raters were experienced with these post-surgery assessment methods. | **NOTE:** In telehealth modality, ROM measurements with the UG were taken with assistance of the subject (no AHP present) by positioning the subject perpendicular to the camera, and using a UG on the computer screen. Field of view and zoom of camera were adjusted as required for telehealth assessments. |
| *14. Hoffmann T, Russell T, Cooke H. et al.(2007)* | Shoulder, elbow, wrist | Seated position; shoulder flexion, shoulder abduction, elbow flexion, wrist flexion, wrist extension, forearm supination and forearm pronation | Other; Internet-based goniometer vs UG | Intra- and inter-rater reliabilities were high; criterion validity also high, including substantial absolute validity (Keogh criteria applied) | N/A | Internet-goniometer measurements were performed using a telerehabilitation system; the axis of the lens of the cameras was visually aligned to the axis of each joint being measured. Anatomical landmarks (same as for UG measurements) were identified on the videoconference image, from which joint angles were calculated by the system. *Sample were stroke patients, and in a clinical (not home) setting. AHP may be required to position patient for accurate telehealth (video) measurement. |
| *15. Russell TG, Jull G & Wootton R. (2003)* | knee | Supine; knee supported on a kneeboard to hold positions; web camera aligned with the joint line of the knee. | Other; Internet-based goniometer (IBG) vs UG (face-to-face) | High agreement was shown between the two devices, establishing validity for the IBG (Keogh standards); Intra-tester reliability was similarly high (by Keogh standards), and was higher than inter-tester reliability. | N/A | The internet-based goniometer relied on still images being taken by the application of the knee in position, identifying landmarks on the images. The software then calculated the knee angle. |

**References**

1. Colaris J, van der Linden M, Selles R, Coene N, Allema JH, Verhaar J. Pronation and supination after forearm fractures in children: reliability of visual estimation and conventional goniometry measurement*. Injury* 2010; **41(6):** 643-6.

2. Croxford P, Jones K, Barker K. Inter-tester comparison between visual estimation and goniometric measurement of ankle dorsiflexion. *Physiotherapy Theory and Practice* 1998; **14(2):** 107-13.

3. Hayes K, Walton JR, Szomor ZR, Murrell GA. Reliability of five methods for assessing shoulder range of motion. *The Australian Journal of Physiotherapy* 2001; **47(4):** 289-94.

4. Higashi M, Shofler D, Manji K, Penera K. Reliability of visual estimation of the first intermetatarsal angle. *The Journal of Foot and Ankle Surgery* 2017; **56(1):** 8-9.

5. Hirsch BP, Webb ML, Bohl DD et al. Improving visual estimates of cervical spine range of motion. *American Journal of Orthopedics* (Belle Mead, NJ) 2014; **43(11):** E261-5.

6. Vauclair F, Aljurayyan A, Abduljabbar FH et al. The smartphone inclinometer: a new tool to determine elbow range of motion? *European Journal of Orthopaedic Surgery & Traumatology: Orthopedie Traumatologie* 2018; **28(3):** 415-21.

7. Youdas JW, Bogard CL, Suman VJ. Reliability of goniometric measurements and visual estimates of ankle joint active range of motion obtained in a clinical setting. *Archives of Physical Medicine and Rehabilitation* 1993; **74(10):** 1113-8.

8. Cabana F, Boissy P, Tousignant M, Moffet H, Corriveau H, Dumais R. Interrater agreement between telerehabilitation and face-to-face clinical outcome measurements for total knee arthroplasty. *Telemedicine and e-Health* 2010; **16**: 293+.

9. Balsalobre-Fernández C, Romero-Franco N, Jiménez-Reyes P. Concurrent validity and reliability of an iPhone app for the measurement of ankle dorsiflexion and inter-limb asymmetries. *Journal of Sports Sciences* 2019; **37(3):** 249-53.

10. Werner BC, Holzgrefe RE, Griffin JW et al. Validation of an innovative method of shoulder range-of-motion measurement using a smartphone clinometer application. *J Shoulder Elbow Surg*. 2014; **23(11)**: e275-e82.

11. Pourahmadi MR, Ebrahimi Takamjani I et al. Reliability and concurrent validity of a new iPhone® goniometric application for measuring active wrist range of motion: a cross-sectional study in asymptomatic subjects. *Journal of Anatomy* 2017; **230(3)**: 484-95.

12. Mejia-Hernandez K, Chang A, Eardley-Harris N, Jaarsma R, Gill TK, McLean JM. Smartphone applications for the evaluation of pathologic shoulder range of motion and shoulder scores—a comparative study. *JSES Open Access* 2018; **2(1)**: 109-14.

13. Cuesta-Vargas AI, Roldan-Jimenez C. Validity and reliability of arm abduction angle measured on smartphone: a cross-sectional study. *BMC Musculoskelet Disord.* 2016; **Feb 20**: 17:93.

14. Hoffmann T, Russell T, Cooke H. Remote measurement via the Internet of upper limb range of motion in people who have had a stroke. *J Telemed Telecare*. 2007;**13(8)**: 401-5.

15. Russell TG, Jull GA, Wootton R. Can the Internet be used as a medium to evaluate knee angle? *Man Ther.* 2003;**8(4)**:242-6.

1. Sorted by primary ROM instrument [↑](#footnote-ref-1)
