# Supplementary material for: Considerations surrounding remote medicolegal assessments: a systematic search and narrative synthesis of the range of motion literature
Source: ANZ J Surg. 2021 Apr 23;92(1-2):46–50. doi: 10.1111/ans.16841 (PMC9291801; doi:10.1111/ans.16841)
Supplement: Supplementary file 5 — Table S2. ROM techniques and technologies (goniometers or Apps)1 with potential to be administered in the vIME setting with assistance of a musculoskeletal‐trained allied health practitioner (AHP) on site. [file ANS-92-46-s005.docx]

**Table S2:** ROM techniques and technologies (goniometers or Apps)^[[1]](#footnote-1)^ with potential to be administered in the vIME setting with *assistance of a musculoskeletal-trained allied health practitioner (AHP) on site*

| *Author(s), year* | Joint(s) | Movement & Position | ROM Instrument(s) | Reliability & validity | Moderating Factors | Limitations, observations, information RE usability in the VIME setting |
| --- | --- | --- | --- | --- | --- | --- |
| *1. Alawna MA; Unver BH; Yuksel EO. (2019)* | ankle | Ankle dorsiflexion - weight bearing lunge; ankle plantar flexion; active | App Type 1; "Smartphone goniometer record" (SGR) App vs Universal goniometer; NOTE: android Samsung Galaxy S3 model | Good inter- and intra-reliability reported when using both App & UG | Requires presence of a health professional to aid placement of the device | The SGR is based on accelerometer technology, is simple to use and reported to be very accurate. It can be used for large joints and also small joints of the hand. Measurement obtained by putting the phone longitudinally on the stationary joint axes to establish the zero point; then moved to the movable joint to determine the angle. Smartphone device needs to be placed on the patient; therefore, would be AHP-assisted in the telehealth setting. |
| *12. Rodriguez-Sanz J; Carrasco-Uribarren A; Cabanillas-Barea S et al. (2019)* | Cervical - spine/neck | Seated position for neck mvts (e.g. lateral flexion, rotation etc) | App Type 1; 2 smartphone apps (“Clinometer” and “Compass”) vs CROM device as criterion | Excellent validity and reliability (intra- and inter-rater) reported for both Apps | Reliability was lower for flexion of upper cervical spine (0.75–0.65) | Smartphone devices did NOT require mounting on a helmet, but placed in a particular position on / beside the patient's head; AHP assistance required to position the device, read the measurement and stabilise the trunk and shoulder girdle to avoid exceeding the cervical spine ROM required |
| *4. Cox RW; Martinez RE; Baker RT et al. (2018)* | ankle | Ankle dorsiflexion; ankle plantar flexion; long sitting position with knee in terminal extension | App Type 1; Clinometer Smartphone Application vs plastic goniometer as criterion | Good validity reported for App; reliability not measured. | Requires presence of a health professional to aid placement of the device | Limited to plantar flexion only (active, not passive); rater needs to be familiar with standard inclinometer procedures; Smartphone device needs to be placed on the patient; therefore AHP assisted in the telehealth setting. |
| *Balsalobre-Fernandez C; Romero-Franco N et al. (2019)* | ankle | Weight-bearing lunge; ankle dorsiflexion | App Type 1; Dorsiflex' iPhone app (iPhone 8 with in-built sensors) vs Professional digital inclinometer | App reported to be highly valid, reliable and accurate (reliability at same level as digital inclinometer). | Need to consider type of smartphone used, i.e. presence of inclination sensors | Larger screen-devices not tested; Need a modern smartphone device with (inbuilt) inclination sensors (iPhone 8 used); Ease of use similar to that of inclinometer, with no additional professional equipment required; app demonstrates correct placement (can be self-administered). |
| *15. Vohralik SL; Bowen AR; Burns J et al. (2015)* | ankle | Weight-bearing lunge test (active); static ankle dorsiflexion | App Type 1; iHandy Level app vs inclinometer | Aadequate reliability and validity established as reported by Keogh et al. (2019) | **Accurate placement** of the smartphone is important and can be challenging for ankle ROM (implies that it requires **instructions** to maximise reliability) | Generalisability limited to active ankle dorsiflexion |
| *20. Mejia-Hernandez K, Chang A, Eardley-Harris N et al.(2018)* | shoulder | Seated, active forward flexion: total abduction, active abduction, and passive abduction. Rotation (internal, external, active, passive) of shoulder in supine. | App Type 1; Inclinometer based smartphone application [GetMyROM], photograph-based [DrGoniometer] smartphone application vs UG and Clinician visual estimation (VE) | Adequate validity & excellent inter-rater reliability demonstrated for both smartphone Apps (intra-rater reliability not measured) (Keogh); **high reliability also shown for VE for the 2 highly trained raters with several years clinical experience** | N/A | iPhone attached to patients' arm at various positions using an arm-band when using the inclinometer-based App; markers on landmarks were required for the photographic-based App; AHP presence for telehealth setting required |
| *5. Guidetti L; Placentino U; Baldari C (2017)* | Cervical - spine/neck | Neck maximal (end-range) movements - frontal flex-extension and left-right side flexion in the sitting position, left-right rotation in the prone position | App Type 1; iPhone 5c smartphone (inclinometric) app vs fluid inclinometer (as criterion) | Reported to be a valid and reliable to measurement of ACROM on the frontal and the sagittal planes | Neck pain may limit the measurement of ACROM (using any device) as measured in the sitting position. | *Smartphone device placed on a helmet worn by the patient; Extends past research showing issues measuring ROM in transverse plane; AHP assistance or an additional supporting tool during movement analysis should be sufficient to assess ACROM in all planes |
| *11. Pourahmadi MR; Bagheri R; Taghipour M et al. (2018)* | cervical spine | Goniometer: active craniocervical; flexion, extension, lateral flexion, and rotation (upright sitting). Smartphone: same as for goniometer; exc for active lateral flexion ROM measured sitting & active cranio-cervical rotation in supine | App Type 1; iPhone app (Goniometer Pro [G-pro]) using an iPhone7 vs UG as criterion | Good to excellent reliability & concurrent validity; exc. inter-rater reliability (Keogh) | N/A | *Active* cranio-cervical ROM Assessment only; AHP assistance would be required in telehealth to position the device and read the measurement; Target used to maintain head posture |
| *22. Pourahmadi MR; Ebrahimi Takamjani I et al. (2017)* | wrist (active ROM) | Seated standard position active wrist ROM, volar/dorsal alignment technique for: flexion, extension, radial deviation & ulnar deviation. | App Type 1; iPhone App (G-pro) comparison for concurrent validity with universal goniometer | Good to excellent intra- and inter-rater reliability (Keogh); although absolute validity was high, relative validity did not quite meet the 'substantial' Keogh threshold. | N/A | Measurement of wrist ROM obtained after strapping the iPhone to the patients' hand (controlling placement and preventing slippage). AHP assistance required in telehealth setting for both devices. |
| *14. Stenneberg MS; Busstra H; Eskes M et al. (2018)* | Cervical - spine/neck | Flexion-extension, lateral bending, rotation; seated position, neutral head-neck start position | App Type 1; iPhone App (with inbuilt sensors) vs electromagnetic tracking device and other basic instruments | Excellent relative validity with absolute validity better for some movements (excellent for right lateral bending) than others (e.g. inadequate for total rotation) (Keogh) | Newer model smartphones will provide most accurate results | iPhone (4S) was fastened securely on the patient's forehead with an iPhone holder with rigid velcro strap. Raters ensured movements executed properly, following the relevant axis. AHP assistance required in telehealth setting |
| *23. Pourahmadi MR, Taghipour M, Jannati E et al. (2016)* | spine - lumbar | Standing lumbar spine flexion & extension | App Type 1; iPhone App 'Tiltmeter' (advanced level and inclinometer) vs gravity-based inclinometer | Good to excellent reliability & concurrent validity (Keogh) | Lumbar spine extension ROM was slightly less reliable when using the iPhone App (difficulty maintaining constant skin contact?). | Raters were experienced orthopaedic physiotherapists; subjects had been familiarised with procedures through demonstration. Strict procedures used for measurement with both devices (e.g. firm placement, accurate spinal landmark detection). AHP assistance required in telehealth setting for both devices. |
| *8. Mehta SP; Barker K; Bowman B et al. (2017)* | knee | Knee flexion and extension; supine position | App Type 1; iPhone goniometer app (i-Goni) vs UG | The 'i-Goni' App demonstrated excellent validity and superior reliability (relative and absolute) than the UG; both showed low SEM (Keogh) | Set of procedures needs to be carefully followed to maximise accuracy; raters were trained in use of both apparatus. | Extended previous studies reliant on healthy participants by using a clinical population; limited generalizability to other joints; both instruments would require AHP assistance in the telehealth setting (anatomical landmark identification). |
| *18. Kolber MJ, Pizzini M, Robinson A et al.(2013)* | spine | Standing position; thoracolumbo-pelvic flexion & extension, isolated lumbar flexion, and thoracolumbar lateral flexion. Standardised positions for AROM. | App Type 1; iPhone inclinometer application (iHandy) vs gravity-based bubble inclinometer | Good intra- and inter-rater reliability reported for both devices (Keogh criteria). Good concurrent validity when using strict measurement procedures. | Devices should not be used interchangeably; required following strict measurement procedures. | Understanding the placement of the iPhone is essential to prevent the screen from rotating at 45 ^o^ (AHP assistance required for implementing standardised positioning in telehealth). |
| *13. Santos C; Pauchard N; Guilloteau A. (2017)* | forearm | Pronation and supination; seated, palms turning towards the floor and then towards the ceiling | App Type 1; iPhone methods (one gyroscope alone with selfie stick) vs two goniometer methods (UG, bubble inclinometer) | Intra-rater reliability was excellent (Keogh criteria); inter-rater also high. | Requires rigorous methodology to ensure reliability; intra-rater reliability slightly lower for pronation than supination overall; measurement instrument / method did not overly affect reliability; selfie-stick method improved reliability of App slightly. Evidence that should not use methods interchangeably. | AHP assistance would be required for telehealth for all 5 methods tested. |
| *6. Jones A, Sealey R, Crowe M et al.(2014)* | knee | Functional weight bearing lunge position on dominant leg | App Type 1; iPhone SG App vs UG | High reported correlation supported concurrent validity for the SG App; measurement error was reported as low for both devices. **Reliability was not measured. | N/A | SG App may be easier to use than UG as landmark identification not required; both instruments would require AHP assistance in the telehealth setting. (Raters both had anatomical knowledge) |
| *32. Banky M; Clark RA; Mentiplay BF et al. (2019)* | Range of joints | Standardised testing positions. Start angle, end angle, full ROM, and peak angular velocity of the relevant joint were recorded. | App Type 1; *Microsoft Kinect* motion sensing device within smartphone vs external 3-D motion-analysis system as criterion; and UG | Smartphone App reported as valid measure of ROM; reliability not reported | Variable hip position impacted ability to measure ROM of knee using smartphone app | Microsoft Kinect showed better accuracy of joint start and end angle than UG; however, requires use of markers (at patient end) and set-up time |
| *Ockendon M & Gilbert RE (2012)* | knee | Knee flexion; supine position; flexion angles between 145 ^o^ hyperextension and 145 ^o^ flexion | App Type 1; novel, accelerometer based, smartphone goniometer technique vs a long-armed conventional goniometer | Yes, adequate reliability and validity established as reported by Keogh. Absolute reliability and validity not reported. | N/A | Both instruments would require AHP assistance in the telehealth setting. |
| *10. Pereira LC; Rwakabayiza S; Lecureux E et al. (2017)* | knee | active and passive measurements in knee flexion and extension; lying supine | App Type 1; smartphone accelerometer-based knee goniometer vs standard goniometer | Excellent relative intra-rater reliability (Keogh); Adequate relative validity also (Keogh) | Knee flexion more reliable than extension | Study sample adult clinical population (not generalisable to healthy adults or to paediatric populations); Devices would require AHP assistance for telehealth setting. |
| *3. Charlton PC; Mentiplay BF; Pua YH et al.(2015)* | hip | Seven hip ROM assessments included: hip flexion, hip abduction and adduction, supine external and internal rotation, and seated hip external and internal rotation. | App Type 1; Smartphone App (Hip ROM Tester, Android operating system, using Samsung Galaxy S2, S3 & Sony Xperia SP) vs bubble inclinometer & 3D motion analysis software | Smartphone app showed good - excellent intra-tester reliability for 4 of 7 movements, and moderate - good for remaining 3 (abduction, adduction and sitting external rotation). Comparable reliability for app and inclinometer. Excellent validity for app relative to 3D analysis system for 6 of 7 movements (exc supine external roation, rated as moderate - good). | *Quality rating was low as judged by Keogh criteria (interpret results with caution) | Measurements recorded by the smartphone at end ROM for each position with phone positioned against the body as per App instructions. (see precise positioning details in paper); Reliability of App comparable to the inclinometer, and methods of use very similar in clinical setting. |
| *9. Milanese S; Gordon S; Buettner P et al., 2014)* | knee | 18 different knee positions; supine position. *Jig placed under right knee standardised knee flexion*; heights provided range of angles. | App Type 1; Smartphone application (the Knee © Goniometer App and a standard universal goniometer | Yes, adequate reliability and validity established as reported by Keogh. Intra-rater reliability was excellent for all examiners, including students. | No evidence for 'experience' influencing reliability of ROM Assessments for either device; instrument reliability deemed to depend on patients remaining still. | Smartphone App may be easier to use than UG as landmark identification not required; both instruments would require AHP assistance in the telehealth setting. |
| *25. Hambly K, Sibley R, Ockendon M. (2012)* | knee | Maximum active knee flexion performed in a supine position | App Type 1; Smartphone application using accelerometer (iGoniometer) vs long-arm goniometer | Acceptable criterion validity for the iGoniometer app (Keogh standards); more research required to establish reliability (Keogh) | N/A | Landmarks identified and marked to facilitate ROM measurement with instruments. |
| *30. Werner BC; Holzgrefe RE; Griffin JW et al. (2014)* | shoulder | Abduction and forward flexion measured standing position; external and internal rotation measured supine | App Type 1; Smartphone clinometer application vs **visual estimation** and goniometer | Excellent inter-rater reliability and average validity reported for the smartphone clinometer (substantially higher than VE); Intra-rater reliability not examined (Keogh) | No evidence for 'experience' (skill level) influencing reliability of ROM Assessments made with the smartphone clinometer | Smartphone held against the patients' arm (varying positions) and measurements read from the screen; likely to require AHP presence for telehealth setting. |
| *2. Behnoush B, Tavakoli N, Bazmi E, et al. (2016)* | elbow | Seated position; active, static flexion, supination and pronation. Goniometer: a pen gripped for supination & pronation; Smartphone: wrist strap was used. | App Type 1; Smartphone inclinometer-based app (bubble inclinometer) on HTC device and UG | Good - excellent *relative* inter-rater reliability (intra-rater reliability not measured); excellent relative concurrent validity, and high absolute validity (Keogh) | N/A | Ease of handling of smartphone noted to be superior to UG instrument; AHP assistance would be required in telehealth |
| *7. Jung SH; Kwon OY; Jeon IC et al. (2018)* | hip / pelvis (lumbo-pelvic stability) | Pelvic transverse rotation angle; dominant leg; Supine position. Performed SLL of dominant leg without bending the knee (see paper for more detail) | App Type 1; Smartphone inclinometer-based App vs 3D motion-analysis system as criterion | Keogh: Validity for pelvic rotation established for the smartphone App; Also established relative and absolute intra-rater reliability (Inter-rater reliability not examined) | N/A | Specific to *active* transverse rotation angle of the pelvis only. Standardised movements used; quite complex frame / apparatus required to fix iPhone positioning. |
| *19. Lim JY, Kim TH, Lee JS. (2015)* | shoulder | Passive ROM of dominant shoulder, horizontal adduction; supine & sidelying position (see paper for details) | App Type 1; Smartphone shoulder ROM measurements in supine vs sidelying positions | Excellent intra-rater reliability for side-lying shoulder ROM; adequate for supine (validity not measured) (Keogh) | Patient positioning was shown to influence reliability of measurements using the smartphone app (side-lying more reliable than standard supine) | Smartphone App measurement would require AHP presence for telehealth setting to assist with device positioning and standardised measurement |
| *17. Furness J, Schram B, Cox AJ (2018)* | spine - thoracic | Thoracic rotation of the spine using the 'seated rotation with bar in front technique'; techniques minimised variations in positioning. | App Type 1; UG and iPhone 6S with Compass App (in-built magnetometer, accelerometer & GPS receiver, previously validated for ROM of the cervical spine) | Good to excellent intra- and inter-rater reliability & concurrent validity, except for absolute validity which did not reach adequacy threshold (Keogh) | Devices should not be used interchangeably. Followed strict measurement procedures. Localised positioning of the iPhone (not possible with the UG) provides “a more uncontam-inated ROM measurement”. Overcomes challenges using UG to measure thoracic ROM. | AHP assistance required in telehealth setting for both devices. |
| *26. Awatani T; Enoki T; Morikita I. (2018)* | ankle | Weight-bearing lunge; ankle dorsiflexion | App Type 2; Smartphone & 'APPmarkerless' i.e. a photographic App^[[2]](#footnote-2)^ vs using virtual goniometer using radiographic images as criterion | High accuracy (Error values of less than 5^o^ reported), reliability and validity | Expertise (e.g. knowledge of anatomy) is important to ensure reliability; positioning of 'APPmarkerless' needs to be precise | Knowledge of anatomy is critical to minimise error; Movement positioning was performed by experienced sport physician - both x-ray and iPhone app measurements were performed while patient held the position. iPhone positioned 2m away to take photograph, phone set perpendicular to the floor and holder, camera lens aligned with the right and left centres and portrait orientation. |
| *28. Otter, S.J., Agalliu, B., Baer, N. et al.(2015)* | Foot - 1st metatarsal phalangeal joint | Standing (on raised platform with prop to maintain joint position); 1st MTPJ (passive) dorsiflexion angle measured. Slight differences in movement instructions for UG and smartphone (see paper). | App Type 2; "Dr G" photographic smartphone application (on Apple iPhone 4S) vs UG as criterion. | Using Keogh criteria: Excellent relative intra-rater reliability for both devices; inter-rater reliability higher for the app than the UG. Concurrent validity not formally reported. | Study excluded anatomical deformity, which may have reduced error and inflated reliability | Dr G app does NOT require anatomical landmark identification (ease-of-use). However, set up was required for each position to standardise and minimise error (i.e. smartphone was cradled in a holder and fixed to a stand, parallel to the floor and same height as the platform that the foot rested on). Study provided further evidence that the use of a robust protocol assists in reducing errors. AHP assistance required if used in telehealth. |
| *29. Reid S & Egan B. (2019)* | forearm | Forearm supination; handheld pencil method. Standing with elbow flexed 90^o^. | App Type 2; "DrGoniometer" App vs UG as criterion | High intra-rater reliability was shown for both the App and UG (Keogh criteria applied). Relative validity was substantial to excellent, using Keogh criteria. | **Standardised instructions,** e.g. consistent distance between phone and forearm | Standardised instructions required for photographic-based app. Photograph could be taken easily and quickly for later analysis using the DrG app. |
| *27. Ferriero G; Vercelli S; Sartorio F et al. (2013)* | knee | Flexion and extension; seated on an isokinetic device with the right leg fixed; right knee placed at different angles. | App Type 2; Smartphone-based application (photographic-based), DrGoniometer (DrG), vs universal goniometer (UG) | *Unable to apply Keogh criteria* to results reported for relative reliability. Absolute reliability was low, not reaching the adequate threshold (Keogh). | Experience shown to improve accuracy using this (photo-based) smartphone app; Set of procedures needs to be carefully followed to maximise accuracy | Only healthy participants used; App was photograph based. Experience improves accuracy; instructions required. |
| *24. Wellmon RH; Gulick DT; Paterson ML et al. (2017)* | Measurement of 3 standardised angles common for hinge joints | Three standardized angles each replicating a hinge joint movement. | App Types 1 & 2; 2 goniometric mobile apps (Goniometer Records, Goniometer Pro), 3 different smartphones (Apple, LG, or Samsung), an inclinometer, and UG | Excellent concurrent validity for the smartphone apps (relative to UG & inclinometer) | Results demonstrate the need to use the same type of phone and same app to maximise (repeat assessment) reliability / minimise error | Angles not measured on humans (purpose-built apparatus with 3 standardised angles) |
| *16. Waddell BS; Duplantier NL; Luo Q et al. (2017)* | knee, hip | Knee and hip immobilized by brace (knee) or bolster (hip) at an angle blinded from the observers. | App Types 1 & 2; 2 smartphone applications (1 virtual inclinometer / photo-based; the other accelerometer-based)vs universal goniometer | Keogh *criteria could not be applied* to interpret reliability and validity | Recommends **using an average of multiple measurements** at a single time, and the **same instrument** to track change in ROM across time | Method of placement and standardisation of measurement techniques were not described. |
| *21. Mitchell K; Gutierrez SB; Sutton S et al. (2014)* | shoulder | Active shoulder external rotation in supine position (See paper for instructions); Raters observed for movement substitutions (e.g. elbow extension, scapular elevation, etc). | App Types 1 & 2; Two smartphone applications: GetMyROM (inclinometer-based) and DrGoniometry (photo-based) vs standard goniometry | Yes, adequate relative intra-rater reliability and excellent relative inter-rater reliability and relative validity established (Keogh). | Experience level did not impact the reliability of the measurements using the devices. | Generalisable to active ER of shoulder (healthy people) only; Expert knowledge of anatomical landmarks required for placement of the DrGoniometry (photo-based) App by an AHP assistant in telehealth setting. |
| *34. Konor, M. M., Morton, S., Eckerson, J. M. et al.(2012)* | Ankle | Dorsiflexion measured in a standing weight-bearing (lunge) position. | Traditional goniometry; standard goniometer, digital inclinometer, and a tape measure. | High intrarater reliability & low standard error of measurement (SEM) for goniometer and digital inclinometer (< 3^o^). | Novice rater able to obtain reliable and accurate measurements with both devices. | Measurements with the instruments taken by a novice rater, and still showed good reliability and low measurement error. Slightly higher reliability for the digital inclinometer. Goniometer stable arm aligned with the floor with mobile arm along the shaft of fibula; inclinometer placed on relevant landmark. AHP-assistance required for telehealth |
| *38. Rome K, Cowieson F. (1996)* | ankle | Active dorsiflexion; supine position with foot placed in positioning block | Traditional Goniometry; universal, fluid, and electro-goniometers | ROM was reported as 'highly reproducible' within raters (intra-rater reliability). Device reliability (absolute, i.e. measurement variability was low) was also high, and better for fluid- and electro-goniometer than the UG. *Keogh criteria could not be applied.* | Rigid protocols required to minimise measurement error. Devices should NOT be used interchangeably. | Ankle positioning block with straps used to secure ankle position. Each of 5 landmarks were identified and marked to aid positioning of the instruments see paper for details RE positioning of instruments). As such AHP required in telehealth setting. |
| *35. Mier CM & Shapiro BS (2013)* | spine, pelvis | Sit and reach test; Soles of both feet flat against the SR box, legs straight. Subject leaned forward without bending the knees. | Other Goniometry; Computer software angle tool that measures thoracic (T), lumbar (L), and pelvic (P) angles (no hand held device required) | Intra-rater reliability was very high for T, L & P angles (slightly lower for lumbar), and SEM very low (Keogh criteria applied); Validity not measured. | N/A | Tool applied during sit-and-reach test (active); Requires an individual well trained in posture and joint ROM assessment to place the marks on the T1, T12, and L5 vertebrae. Captured on video and later assessed by the computer software angle tool. Digital camera set-up required, as well as sit and reach testing positioning apparatus). |
| *33. Carey MA, Laird DE, Murray KA etal. (2010)* | Elbow, shoulder | Active elbow flexion; active internal rotation; passive and active external shoulder rotation (no position information) | Other Goniometry; Digital goniometer vs standard UG | Adequate concurrent and criterion validity demonstrated for the DG, with equivalent intra-rater reliability to the UG. | N/A | "Novel" (2010) features of the DG may make it preferable to use by clinicians (e.g. horizontal and vertical bubble levels for planar alignment, digital display, one-handed operation. Would need to be AHP assisted if used in telehealth. |
| *36. Sidaway B; Euloth T; Caron H et al.(2012)* | ankle | Ankle dorsiflexion; lunge with knee extension | Other goniometry; i.e. open-chained goniometry, closed-chained goniometry, inclinometry, and a novel trigonometric technique vs computerised video analysis as criterion | Excellent reliability & validity reported for the new trigonometric technique | N/A | Training and a set of standardised instructions required to use the novel trigonometric technique (using tape measure and ruler); use of instruments would require a (trained) person to be present with the patient |
| *37. Szekeres M, MacDermid J, Birmingham T et al.(2016)* | forearm | Seated position; forearm rotation (elbow at side, humerus vertical orientation); active pronation and supination | Other Goniometry; modified finger goniometer (MFG) vs standard goniometer | Inter-rater reliability slightly higher for the MFG; Intra-rater not measured. | MFG method of forearm rotation offers some advantages in minimising error, as it uses bony landmarks for placement and a plumb line to determine vertical orientation. | Both methods would require AHP assistance for use in telehealth setting |
| *31. Armstrong AD, MacDermid JC, Chinchalkar S et al. (1998)* | forearm, elbow | Seated and positioned in a standardised manner for flexion & extension movements, with elbow flexion for supination and pronation | Other Goniometry; Universal goniometer, computerised goniometer (Super Pro goniometer), mechanical rotation measuring device | Intra-rater reliability was high for all three instruments (Keogh criteria applied); Intra-tester error also low, but slightly higher for forearm rotation than flexion or extension. | Level of experience of examiner did not influence reliability; same examiner and same instrument minimises error of measurement. Type of instrument did not significantly influence reliability. | Standardised methods likely improved the consistency of measurements. Raters would need training to use the more unfamiliar goniometers examined by this study. Standard positions used, no technical apparatus required to aid positioning. AHP required to assist goniometer measurements in telehealth setting. |

**References**

1. Alawna MA, Unver BH, Yuksel EO. The reliability of a smartphone goniometer application compared with a traditional goniometer for measuring ankle joint range of motion. *Journal of the American Podiatric Medical Association* 2019; **109(1)**: 22-9.

2. Behnoush B, Tavakoli N, Bazmi E et al. Smartphone and universal goniometer for measurement of elbow joint motions: a comparative study. *Asian Journal of Sports Medicine* 2016; **7(2)**: e30668.

3. Charlton PC, Mentiplay BF, Pua Y-H, Clark RA. Reliability and concurrent validity of a smartphone, bubble inclinometer and motion analysis system for measurement of hip joint range of motion. *Journal of Science and Medicine in Sport* 2015; **18(3)**: 262-7.

4. Cox RW, Martinez RE, Baker RT, Warren L. Validity of a smartphone application for measuring ankle plantar flexion. *Journal of Sport Rehabilitation* 2018; **27(3)**: 1-N.PAG.

5. Guidetti L, Placentino U, Baldari C. Reliability and criterion validity of the smartphone inclinometer application to quantify cervical spine mobility. *Clinical Spine Surgery* 2017; **30(10)**: E1359-e66.

6. Jones A, Sealey R, Crowe M, Gordon S. Concurrent validity and reliability of the simple goniometer iPhone app compared with the universal goniometer. *Physiother Theory Pract*. 2014; **30(7)**: 512-6.

7. Jung S-h, Kwon O-y, Jeon I-c, Hwang U-j, Weon J-h. Reliability and criterion validity of measurements using a smart phone-based measurement tool for the transverse rotation angle of the pelvis during single-leg lifting. *Physiother Theory Pract*. 2018; **34(1)**: 58-65.

8. Mehta SP, Barker K, Bowman B, Galloway H, Oliashirazi N, Oliashirazi A. Reliability, concurrent validity, and minimal detectable change for iPhone goniometer app in assessing knee range of motion. *The Journal of Knee Surgery* 2017; **30(6)**: 577-84.

9. Milanese S, Gordon S, Buettner P et al. Reliability and concurrent validity of knee angle measurement: smart phone app versus universal goniometer used by experienced and novice clinicians. *Manual Therapy*. 2014; **19(6)**: 569-74.

10. Pereira LC, Rwakabayiza S, Lecureux E, Jolles BM. Reliability of the knee smartphone-application goniometer in the acute orthopedic setting. *The Journal of Knee Surgery* 2017; **30(3)**: 223-30.

11. Pourahmadi MR, Bagheri R, Taghipour M, Takamjani IE, Sarrafzadeh J, Mohseni-Bandpei MA. A new iPhone application for measuring active craniocervical range of motion in patients with non-specific neck pain: a reliability and validity study. *The Spine Journal* 2018; **18(3)**: 447-57.

12. Rodriguez-Sanz J, Carrasco-Uribarren A, Cabanillas-Barea S et al. Validity and reliability of two smartphone applications to measure the lower and upper cervical spine range of motion in subjects with chronic cervical pain. *Journal of Back and Musculoskeletal Rehabilitation* 2019; **32(4)**: 619-27.

13. Santos C, Pauchard N, Guilloteau A. Reliability assessment of measuring active wrist pronation and supination range of motion with a smartphone. *Hand Surgery and Rehabilitation.* 2017; **36(5)**: 338-45.

14. Stenneberg MS, Busstra H, Eskes M et al. Concurrent validity and interrater reliability of a new smartphone application to assess 3D active cervical range of motion in patients with neck pain. *Musculoskeletal Science and Practice* 2018; **34**: 59-65.

15. Vohralik SL, Bowen AR, Burns J, Hiller CE, Nightingale EJ. Reliability and validity of a smartphone app to measure joint range. *American Journal of Physical Medicine & Rehabilitation* 2015; **94(4)**: 325-30.

16. Waddell BS, Duplantier NL, Luo Q, Meyer MS, Duncan SFM. Smartphone-based goniometry accuracy in clinical scenarios. *Journal of Surgical Orthopaedic Advances* 2017; **26(4)**: 223-6.

17. Furness J, Schram B, Cox AJ, Anderson SL, Keogh J. Reliability and concurrent validity of the iPhone((R)) Compass application to measure thoracic rotation range of motion (ROM) in healthy participants. *PeerJ.* 2018; **6**: e4431.

18. Kolber MJ, Pizzini M, Robinson A, Yanez D, Hanney WJ. The reliability and concurrent validity of measurements used to quantify lumbar spine mobility: an analysis of an iPhone(R) application and gravity based inclinometry*. Int J Sports Phys Ther*. 2013; **8(2)**: 129-37.

19. Lim J-Y, Kim T-H, Lee J-S. Reliability of measuring the passive range of shoulder horizontal adduction using a smartphone in the supine versus the side-lying position. *Journal of Physical Therapy Science* 2015; **27(10)**: 3119-22.

20. Mejia-Hernandez K, Chang A, Eardley-Harris N, Jaarsma R, Gill TK, McLean JM. Smartphone applications for the evaluation of pathologic shoulder range of motion and shoulder scores—a comparative study. *JSES Open Access* 2018; **2(1)**: 109-14.

21. Mitchell K, Gutierrez SB, Sutton S, Morton S, Morgenthaler A. Reliability and validity of goniometric iPhone applications for the assessment of active shoulder external rotation. *Physiotherapy Theory and Practice* 2014; **30(7)**: 521-5.

22. Pourahmadi MR, Ebrahimi Takamjani I et al. Reliability and concurrent validity of a new iPhone® goniometric application for measuring active wrist range of motion: a cross-sectional study in asymptomatic subjects. *Journal of Anatomy* 2017; **230(3)**: 484-95.

23. Pourahmadi MR, Taghipour M, Jannati E, Mohseni-Bandpei MA, Ebrahimi Takamjani I, Rajabzadeh F. Reliability and validity of an iPhone(®) application for the measurement of lumbar spine flexion and extension range of motion. *PeerJ*. 2016; **4**: e2355.

24. Wellmon RH, Guliek DT, Paterson ML, Guliek CN. Validity and reliability of 2 goniometrie mobile apps: device, application, and examiner factors. *Journal of Sport Rehabilitation* 2016; **25(4)**: 371-9.

25. Hambly K, Sibly R, Ockendon M. Level of agreement between a novel smartphone application and a long arm goniometer for the assessment of maximum active knee flexion by an inexperienced tester. *International Journal of Physiotherapy and Rehabilitation* 2012; **2**: 1-14.

26. Awatani T, Enoki T, Morikita I. Inter-rater reliability and validity of angle measurements using smartphone applications for weight-bearing ankle dorsiflexion range of motion measurements. *PhysicalT in Sport : Official Journal of the Association of Chartered Physiotherapists in Sports Medicine* 2018; **34**: 113-20.

27. Ferriero G, Vercelli S, Sartorio F et al. Reliability of a smartphone-based goniometer for knee joint goniometry. *International Journal of Rehabilitation Research Internationale* 2013; **36(2)**: 146-51.

28. Otter SJ, Agalliu B, Baer N et al. The reliability of a smartphone goniometer application compared with a traditional goniometer for measuring first metatarsophalangeal joint dorsiflexion. *Journal of Foot and Ankle Research* 2015; **8(1)** : 30.

29. Reid S, Egan B. The validity and reliability of DrGoniometer, a smartphone application, for measuring forearm supination. *Journal of Hand Therapy* 2019; **32(1)**: 110-7.

30. Werner BC, Holzgrefe RE, Griffin JW et al. Validation of an innovative method of shoulder range-of-motion measurement using a smartphone clinometer application. *J Shoulder Elbow Surg*. 2014; **23(11)**: e275-e82.

31. Armstrong AD, MacDermid JC, Chinchalkar S, Stevens RS, King GJ. Reliability of range-of-motion measurement in the elbow and forearm. *J Shoulder Elbow Surg*. 1998; **7(6)**: 573-80.

32. Banky M, Clark RA, Mentiplay BF, Olver JH, Kahn MB, Williams G. Toward accurate clinical spasticity assessment: validation of movement speed and joint angle assessments using smartphones and camera tracking. *Archives of Physical Medicine and Rehabilitation* 2019; **100(8)**: 1482-91.

33. Carey MA, Laird DE, Murray KA, Stevenson JR. Reliability, validity, and clinical usability of a digital goniometer. *Work* 2010; **36(1)**: 55-66.

34. Konor MM, Morton S, Eckerson JM, Grindstaff TL. Reliability of three measures of ankle dorsiflexion range of motion. *Int J Sports Phys Ther*. 2012; **7(3)**: 279-87.

35. Mier CM, Shapiro BS. Reliability of a computer software angle tool for measuring spine and pelvic flexibility during the sit-and-reach test. *Journal of Strength and Conditioning Research* 2013; **27(2)**: 501-6.

36. Sidaway B, Euloth T, Caron H, Piskura M, Clancy J, Aide A. Comparing the reliability of a trigonometric technique to goniometry and inclinometry in measuring ankle dorsiflexion. *Gait & Posture* 2012; **36(3)**: 335-9.

37. Szekeres M, MacDermid JC, Birmingham T, Grewal R. The inter-rater reliability of the modified finger goniometer for measuring forearm rotation. *Journal of Hand Therapy : Official Journal of the American Society of Hand Therapists* 2016; **29(3):** 292-8.

38. Rome K, Cowieson F. A reliability study of the universal goniometer, fluid goniometer, and electrogoniometer for the measurement of ankle dorsiflexion. *Foot & Ankle International* 1996; **17(1):** 28-32.

1. Sorted by primary ROM instrument [↑](#footnote-ref-1)
2. Note - the majority of photographic-based apps were excluded from this review. Saving photos for later examination raises confidentiality and consent issues, as well as viability issues ('framing', positioning etc) requiring require precise instructions differing for each joint and position. Footnote attached to Awatani paper [↑](#footnote-ref-2)
